# The Experience of Long COVID Among American Indian Individuals in Three Great Plains Communities

**DOI:** 10.1007/s40615-025-02618-z

**Published:** 2025-09-04

**Authors:** Matthew Tobey, Sara J. Purvis, Bethany-Rose Daubman, Mary J. Isaacson, Tinka Duran, Gina Johnson, J. R. LaPlante, Katrina Armstrong

**Affiliations:** 1 Department of Medicine, Massachusetts General Hospital, 100 Cambridge Street, 16th Floor, Boston, MA 02114, USA; 2 Division of Palliative Care and Geriatric Medicine, Massachusetts General Hospital, Boston, MA 02114, USA; 3 College of Nursing South, Dakota State University, Rapid City, SD, USA; 4 Great Plains Tribal Epidemiology Center, Great Plains Tribal Leaders’ Health Board, Rapid City, SD, USA; 5 Community Health Prevention Programs, Great Plains Tribal Leaders’ Health Board, Rapid City, SD, USA; 6 American Indian Health Initiative, Avera Health, Sioux Falls, SD, USA; 7 Columbia Irving Medical Center, New York City, NY, USA

**Keywords:** COVID, American Indian, Tribal, Rural

## Abstract

Long COVID may impact populations differently. In July 2023, at the direction of a community advisory board, we administered a cross-sectional survey to explore attitudes and experiences of long COVID among members of three American Indian Reservation communities in the Great Plains. Just over half of the 843 respondents considered long COVID to be an important issue in their community, an attitude that was associated with younger age, identifying as male, having more than a high school education, full-time employment, living with children, and living on the Reservation. Of survey respondents who reported having had COVID-19, 40% reported ongoing symptoms at the time of the survey. Having ongoing symptoms was associated with identifying as female, living alone with children, and having had long COVID-19 symptoms during the initial infection. To explore ongoing symptoms, we performed a factor analysis that identified three symptom clusters with distinct sociodemographic associations. The results suggest that health and workforce sequelae of COVID-19 infections may present challenges for the surveyed communities and that ongoing symptoms after COVID-19 infection were common.

## Introduction

Beginning in 2020, healthcare professions and researchers have been learning more about the COVID-19 virus and its long-term impacts. A large percent of the population recovers from COVID-19 within a few days or weeks, but for some, their symptoms last for months or longer. This is Long COVID which per the National Academies of Sciences, Engineering, and Medicine, “is an infection-associated chronic condition that occurs after SARS-CoV-2 infection and is present for at least 3 months as a continuous, relapsing and remitting, or progressive disease state that affects one or more organ systems” [1]. The National Center for Health Statistics’ House Pulse Survey estimates a national prevalence of long COVID of 18%, defined as, “did you have any symptoms lasting 3 months or longer that you did not have prior to having coronavirus or COVID-19” [2]. The same survey estimates that 4% percent of US adults report having activity limitations due to long COVID, and 1% report significant activity limitations [2]. Experiencing long COVID has been associated with biological findings, including increased immunological cell activation, presence of autoantibodies, persistence of COVID-19, reactivation of other viruses, and a decreased COVID-19 specific immune response [3, 4]. Among those with long COVID, dozens of symptoms have been reported, the most frequent being difficulty concentrating, general functional impairment, fatigue, and muscle weakness [4–6]. Symptom clusters vary between studied populations [7, 8].

Of the 9.7 million individuals who identify as American Indian and Alaska Native (AIAN) alone or in combination with another race, 1.2 million live on the 324 AI Reservations in the United States [9]. AIAN individuals, compared with non-Hispanic White persons, have had rates of COVID-19 cases, hospitalizations, and deaths higher by 1.6, 2.4, and 2.0 times, respectively [10]. Long COVID was self-reported more frequently among AIAN individuals and rural dwellers in a nationwide adjusted analysis [11].

In the Great Plains, there are 18 Ai Reservations where health care is provided by the Indian Health Service, Tribes, and the private sector. As of October 2021, an electronic survey found that, of 679 respondents, 83% had received a COVID-19 test, 32% had tested positive for COVID-19, and 85% had received at least one COVID-19 vaccine dose [12]. At that time, the national rate of receipt of at least one vaccine dose among AIAN individuals was between 55 and 60% [13].

In July 2023, based on a request from a COVID-19 related community advisory board (CAB) comprised of members of three Great Plains AI Tribes, we conducted a survey to characterize the local experience of long COVID.

## Methods

In 2020, our team established a CAB with members selected based on experience with research programs, longstanding residence in their communities, and engagement with their community’s health systems. A prior survey had reported on attitudes and behaviors regarding COVID-19 testing and vaccination [12, 14].

We developed an eleven-item questionnaire (Appendix) to address the CAB’s request to investigate the local experience with and impact of long COVID. The eleven questions included nine questions from the Longitudinal Population Studies COVID-19 questionnaire, a tool used by several studies to assess COVID-19 symptoms [15], and two questions about the impact of COVID: whether the respondent knew someone with long COVID; and whether long COVID was a major issue in their community. Participants were required to be 18 years or older and identify as an enrolled member of a federally recognized Tribe.

On July 19th, 2023, we posted a link to the survey with a flier describing the survey on the Facebook page of the Great Plains Tribal Leaders Health Board. We exclusively administered the survey online, using REDCap^®^. In a previous survey, we distributed the survey through both Facebook and paper methods; however, the paper surveys did not significantly improve participation, so the researchers opted to focus on Facebook this time as this is a primary means of information and event sharing in the communities. To minimize the risk of illegitimate responses, the study team reviewed and minimally filtered responses based on the number of questions that were completed (i.e., if no responses were reported or only the first few questions) and if REDCap reported the survey response as complete. As participants were required to identify as an enrolled member of a federally recognized Tribe, responses that did not select a Tribal affiliation were removed.

The survey enrolled 843 individuals on the same day it opened, exceeding our enrollment target of 650 individuals, and was thus closed. Survey participants completed a consent form, the survey, and then a separate form to receive a $25 VISA gift card by email or mail. The consent and gift card form responses were recorded separately from the survey, which was anonymous.

All analyses were conducted in STATA 15. To assess the association between respondent characteristics and a perception that long COVID was a major issue in the community, we performed three analyses. First, we used Wilcoxon ranked sum tests to assess whether respondent characteristics were associated with that perception. We also examined the association for each respondent characteristic category using odds ratios and 95% confidence intervals (CI) with chi-squared testing for statistical significance. Third, we performed a logistic regression including age, gender, education, household composition, employment, living on a Reservation, and the personal length of COVID-19 symptoms.

**Fig. 1:**
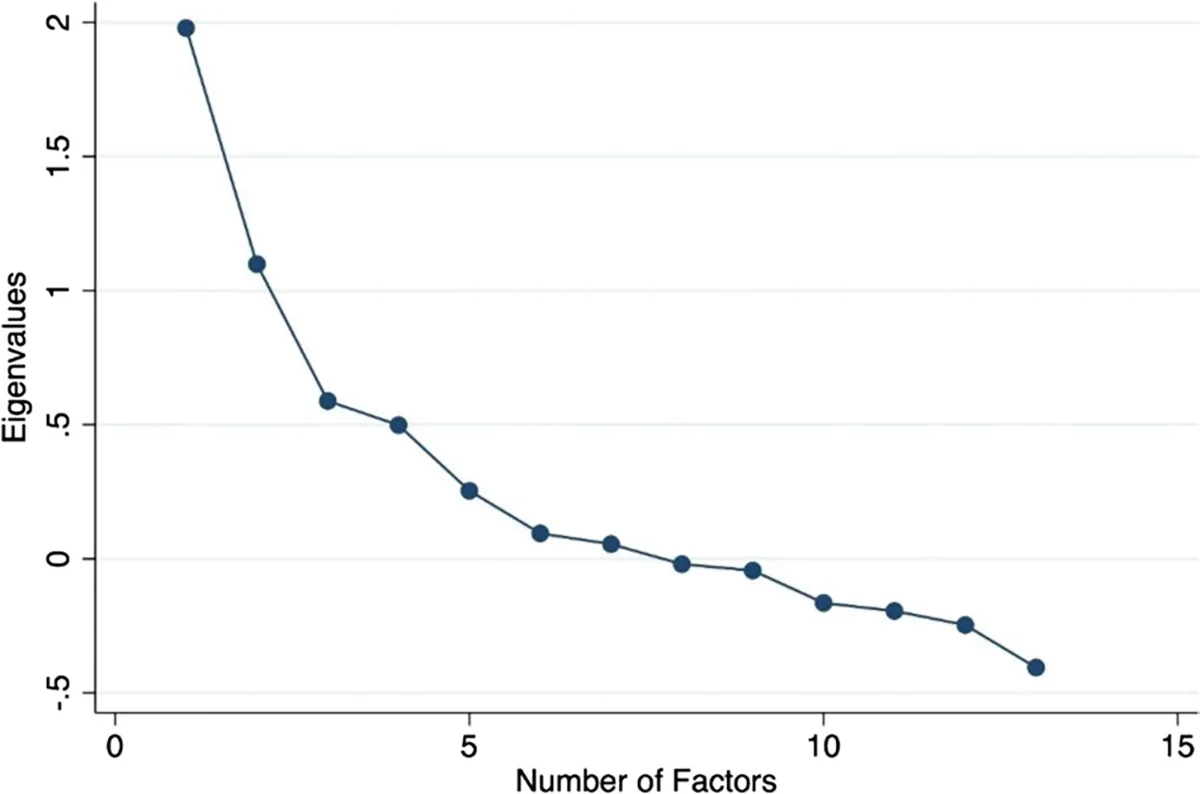
Factor analysis, scree plot of eigenvalues

To assess the persistence and characteristics of COVID-19 symptoms, we performed three analyses. First, we assessed which respondent characteristics were associated with not feeling back to normal after COVID-19 infection at the time of the survey. Given the small number of participants who reported not being back to normal after COVID-19 infection, we reduced the number of independent variables included in the regression analysis to maximize the model fit. Variables were included using a forward selection process with age category being forced into the model as a continuous variable. Second, we performed a factor analysis to identify symptom clusters among the 13 symptoms. We retained three factors with Eigenvalues greater than 1 guided by the scree plot (Fig. 1), with all symptoms other than heart problems and mood problems having loadings greater than 0.3 on one of the factors. Third, we used linear regression models to assess associations with each symptom cluster.

The study was reviewed and considered exempt from Institutional Review Board (IRB) review by the Massachusetts General Brigham IRB. The study was also reviewed and approved by the Great Plains IRB and all three Tribes via Tribal IRBs or Tribal Councils. The identities of the three Tribes are suppressed for privacy. A plain-language summary of findings has been shared with the communities, and all Tribal Councils or Tribal IRBs, as applicable, received a pre-publication draft of this manuscript for review, as did the Great Plains IRB.

## Results

The survey was completed by 843 people completed the survey. Participant characteristics are shown in Table 1. Nearly half of participants identified as male, and slightly over half as female. Two participants identified as non-binary/gender non-conforming. Twenty-five percent of participants had not completed high school and 31% had a high school degree only. Nearly half lived on a Tribal Reservation. Eighteen percent of participants lived alone, 24% lived with a partner only, 43% lived with a partner and children, and 10% lived with children only.

Nearly 60% of participants reported knowing someone with long COVID and 54% said it was a major issue in their community. In bivariate analyses, believing long COVID was a major issue was significantly associated with younger age (p ≤ 0.01), more than a high school education (p ≤ 0.01), identifying as male (p ≤ 0.01), being employed full-time (p ≤ 0.01), living with children (p ≤ 0.01), and living on the Reservation (p ≤ 0.01). The length of symptoms from COVID-19 (p ≤ 0.01) and knowing someone with long COVID (p ≤ 0.01) were also associated with the belief that long COVID was a major issue (Table 1).

After multivariate adjustment, believing that long COVID was a major issue in the community remained significantly associated with having attended some college or technical school (OR = 1.71; 95% CI [1.11, 2.63]), living with a partner and children (OR = 1.84; 95% CI [1.17, 2.88]), being employed full-time (Part-time: OR = 0.49; 95% CI [0.33, 0.73]; Seasonal: OR = 0.37; 95% CI [0.23, 0.61]), and having had a longer duration of COVID-19 symptoms (2–3 Weeks: (OR = 3.42; 95% CI [1.71, 6.85]; 4 + Weeks: OR = 5.18; 95% CI [2.06, 13.04]). Associations with age, gender, and living on a Reservation were no longer statistically significant (Table 2).

Twenty percent of respondents reported having had COVID-19 themselves (n = 173), and an additional 10% (n = 81) were unsure if they had had COVID-19. Among those who reported having had COVID-19, 7% reported not being back to normal and 25% had had symptoms for 4 or more weeks. In multivariate regression, not being back to normal after COVID-19 was inversely associated with identifying as male (OR = 0.03, 95% [0.00, 0.38]), living with children alone (OR = 30.7, 95% [1.67, 565.4]), and being sick for four weeks or more with the initial illness (OR = 14.4, 95% [1.84, 112]) (Table 3).

Of note, although only 7% of participants said they were not back to normal, a greater percentage endorsed a range of 13 symptoms 12 or more weeks after having COVID-19, ranging from 40% reporting problems thinking and communicating to 2% reporting skin rashes. Factor analysis revealed three symptom clusters, as shown in Table 4. These three symptom clusters had varying associations with sociodemographic patient characteristics (Table 5). Having more symptoms in the breathing/muscle/sleeping cluster was associated with being 25 to 34 years of age (r^2^ = 0.13; 95% CI [0.05, 0.20]), identifying as male (r^2^ = 0.07; 95% CI [0.00, 0.13]), living with a partner (r^2^ = 0.12; 95% CI [0.03, 0.22]) or living with a partner and children (r^2^ = 0.16; 95% CI [0.07, 0.24]), and working part-time (r^2^ = 0.24; 95% CI [0.15, 0.34]) or being unemployed/self-employed (r^2^ = 0.15; 95% CI [0.04, 0.26]). Having more symptoms in the concentration/mood cluster were associated with being 35 to 44 years of age (r^2^ = −0.13; 95% CI [−0.22, −0.03]), identifying as female (male: r ^2^ = −0.15; 95% CI [−0.23, −0.07]), living with a partner (r^2^ = 0.20; 95% CI [0.08, 0.32]), partner and children (r^2^ = 0.39; 95% CI [0.29, 0.49]), children only (r^2^ = 0.05; 95% CI [0.00, 0.25]), or other family members (r^2^ = 0.15; 95% CI [0.02, 0.28]), having greater than a college education (r^2^ = −0.24; 95% CI [−0.40, −0.07]), and being employed (unemployed: r^2^ = −0.15; 95% CI [−0.28, −0.01]). Having more symptoms in the altered smell/dizziness cluster was only associated with living with a partner (r^2^ = 0.23; 95% CI [0.04, 0.42]), partner and children (r^2^ = 0.27; 95% CI [0.11, 0.44]), or children (r^2^ = 0.24; 95% CI [0.03, 0.45]) (Table 6).

## Discussion

This study reports the perceptions and experiences of long COVID in Tribal communities in the US, a critically important and understudied topic. The results offer important insights into the burden of long COVID in these communities and the factors that may influence the likelihood of experiencing patterns of symptoms.

First, long COVID was perceived as an important issue by more than half of study participants from the three Tribal communities, particularly among younger individuals with higher levels of education, those who had had a prolonged course of COVID-19 infection, and those with full-time employment. Long COVID was also perceived as important by our CAB, who requested this study due to the condition’s negative impact on the local workforce. Few studies have examined public attitudes about long COVID, though a high proportion of individuals with a diagnosis of long COVID have been noted to perceive stigma about the diagnosis [16, 17]. Long COVID has been noted to cause substantial workforce impacts, including reductions in workforce participation and reduced hours worked [18, 19]. These impacts may be amplified in rural Reservation-based communities, which have high rates of work in the public sector and fewer workers with a bachelor’s degree or higher [20, 21]. If reductions in the workforce caused by long COVID create challenges in sustaining local businesses and public sector programming, then educated, full-time workers may be more likely to interact with such challenges, and thus to consider long COVID to have an important impact.

Second, seven percent of individuals reported not being back to normal after COVID-19 infection and 40% had ongoing symptoms. These findings are in line with other studies, such as CDC’s Household Pulse Survey, that last July reported active long COVID at 5.8%, and ever having had long COVID at 15.4% [2]. We found ongoing symptoms to be strongly associated with being a woman, living with children without other household members, and having had a longer symptomatic period from the initial infection. These findings align with those of other reports, including higher self-reported rates of long COVID among individuals identifying as female and those with more severe initial COVID-19 symptoms [11, 22, 23], and also raise the potential impact of living alone with children on the incidence or management of persistent COVID-19 symptoms. Individuals with solo responsibility for children may be less likely to be able to undertake activities to manage or improve symptoms of long COVID, a process that is well recognized to involve multiple barriers [24]. Activities to manage or improve symptoms of long COVID include visiting a clinician, active recovery, attending support groups, receiving mental health support, negotiating for accommodations in school or the workplace, and other psychosocial support [17, 25]. These services may be limited in Tribal and rural areas. Similarly, the demands of providing childcare by oneself could lead respondents to be more aware of long COVID symptoms and thus report them.

Third, 40% of respondents reported ongoing symptoms at 12 weeks after infection. Their symptoms aggregated into three distinct clusters. The first symptom cluster, with breathing, muscle aches, and trouble sleeping, was more common among younger, partnered men who were not employed full-time. These demographics suggest a subpopulation that may experience limitations related to intermittent manual labor and exertion, either for employment or household commitments. The second symptom cluster, with concentration and mood symptoms, was associated with being 35 to 44 years of age, identifying as female and living with a partner or other family members, as well as having greater than a college education and being employed. While multiple studies have demonstrated associations between gender identity and long COVID symptoms, these results suggest that cognitive symptoms may also be more common in those who have greater cognitive demands at work, although we did not measure specific job tasks or requirements. The third cluster, with altered smell and dizziness, had fewer demographic associations. These results add to the literature demonstrating that long COVID symptoms and symptom clusters vary across populations, emphasizing the importance of community-based research studies for understanding the lived experiences of different groups [7, 8]. For instance, long COVID studies that enroll patients at long COVID clinics are likely to find different symptom patterns than our Tribal community-based sample [25, 26].

Fourth, this survey provides an additional estimate of rates of COVID-19 infection in this population, with 30% of respondents in July 2023 stating they had a past definite or possible COVID-19 infection. In a similar survey we administered in October 2021, self-reported rates of a positive COVID-19 test were 32% [14]. In June – August 2023, the Household Pulse Survey, also a 20-min online survey, found South Dakota’s self-reported rate of past COVID-19 infection to be 55%, asking if the individuals either had a positive test or had been informed of a COVID-19 diagnosis by a clinician [2]. Lower estimates in this study’s survey may reflect lower rates of infection among respondents, differences in sampling strategies between the surveys, and/or individuals with asymptomatic or mild COVID-19 infection being more likely to report positive COVID-19 tests than COVID-19 infections. COVID-19 vaccination through the Indian Health Service was widespread by February 2021 and may have led to lower rates of infection in these communities [27].

Finally, our study highlights the complexity of measuring and defining long COVID, particularly in survey studies. Surveys relying on self-reporting provide less consistent rates of initial COVID-19 infection compared with studies that include testing results. The large difference in the rates of respondents who reported long COVID (7%) and those who reported ongoing symptoms (40%) was notable. The ongoing burden of symptoms may relate, for instance, to high rates of chronic conditions [28, 29]. There might also have been reluctance to attribute symptoms to long COVID due to stigma associated with the condition.

Limitations of this study include that its online-only nature may have limited participation by individuals lacking broadband access. In a prior survey in the same population, paper survey distribution did not substantially increase participation [12, 14] The gender and age distribution of our participants is similar to that of the AI/AN population within the Great Plains Area (Table 7) [30]. However, the distribution of participant’s age and unemployment differ. Recruiting only on Facebook may have led to selection bias and could affect the prevalence estimates and symptom cluster distributions.

In addition, symptom reporting was not linked to a clinical verification or test-confirmed COVID-19 which may affect both the denominator (i.e., who had COVID-19) and the outcome (i.e., who had long COVID). This data is not representative of all AIAN reservation-based communities and the majority of AIAN individuals now reside in urban areas. The geographical focus limits the generalizability to other communities; however, the researchers described the sample, setting, and methods for applicability.

Given the nature of long COVID symptoms, results can be sensitive to survey language, and comparisons with other studies and samples should be made accordingly. For instance, comparison should be interpreted with caution with studies that linked survey results to testing or biological data. Finally, individuals suffering from long COVID could have had difficulty completing a 30-min survey.

Further qualitative research, such as through talking circles, an Indigenous qualitative research technique, could provide more detailed information about the experience and importance of long COVID in this population. There is a lack of research on the impacts of long COVID among AIAN communities and more work must be done. Healthcare professionals and policy makers must consider that community members are impacted by long COVID and assist in implementing supportive activities for these community members. Understanding the prevalence of long COVID and how it impacts community members is essential for providing culturally responsive care and promoting healthy communities.

## Conclusion

We found that, in summer 2023, long COVID was perceived to be an important issue among study participants living in three rural-Reservation-based Tribes in the Great Plains, especially by individuals with full-time employment, those who lived on a Reservation, and those who had themselves experienced longer-duration COVID-19 symptoms.

## Supplementary Material

Appendix

**Supplementary Information** The online version contains supplementary material available at https://doi.org/10.1007/s40615-025-02618-z.

## Figures and Tables

**Table 1:** Survey respondent characteristics and whether Long COVID was perceived as a major issue

	Total	Long COVID is a major issue		
		Yes	No	*p*-value

Age	n (%)	n (%)	n (%)	
18–24	0 (0)	0 (0)	0 (0)	0.01*
25–34	326 (38.7)	180 (52.5)	144 (41.2)	
35–44	305 (36.2)	178 (58.4)	127 (41.6)	
45 or older	212 (25.2)	95 (44.8)	117 (55.2)	
Male (vs female)	387 (46.0)	227 (58.7)	159 (41.1)	0.01*
	454 (54.0)	224 (49.3)	229 (50.4)	
Educational attainment				
Less than high school degree	192 (23.3)	87 (45.3)	105 (54.7)	0.00*
High school degree	262 (31.8)	119 (45.4)	143 (54.6)	
Some college or technical school	271 (32.9)	184 (67.9)	86 (31.7)	
College degree or higher	99 (12.0)	53 (53.5)	44 (44.4)	
Household composition				
Alone	144 (17.3)	61 (42.4)	83 (57.6)	0.00*
Partner only	197 (23.6)	94 (47.7)	102 (51.8)	
Partner and children	344 (41.2)	205 (59.6)	139 (40.4)	
Children only	60 (7.19)	29 (48.3)	30 (50.0)	
Other	90 (10.8)	59 (65.6)	31 (34.4)	
Employment				
Full-time	345 (41.0)	240 (69.6)	103 (29.9)	0.00*
Part-time	342 (40.7)	150 (43.9)	192 (56.1)	
Seasonal	128 (15.2)	48 (37.5)	80 (62.5)	
Unemployed/self-employed	26 (3.09)	13 (50.0)	13 (50.0)	
Lives on a Reservation (vs not)	391 (46.4)	229 (58.6)	161 (41.2)	0.00*
Length of COVID-19 symptoms				
No COVID-19	588 (78.7)	292 (49.7)	295 (50.2)	0.00*
Less than 2 weeks	37 (4.95)	28 (75.7)	9 (24.3)	
2–3 weeks	82 (11.0)	67 (81.7)	14 (17.1)	
4 or more weeks	40 (5.35)	33 (82.5)	7 (17.5)	
Know someone with long COVID	497 (59.1)	306 (61.6)	190 (38.2)	0.00*
(vs not)	344 (40.9)	146 (42.4)	198 (57.6)	
Long COVID a major issue	453 (53.7)	–	–	–

* p ≤ 0.05

**Table 2:** Perception that Long COVID is a major issue, by respondent characteristics

	Odds Ratio	*p*-value	95% Confidence Interval	

Age				
18–24	Reference			
25–34	1.35	0.10	0.94	1.93
35–44	1.13	0.60	0.72	1.78
45 or older	1.42	0.43	0.60	3.36
Male (vs female)	1.08	0.66	0.77	1.51
Educational attainment				
Less than high school degree	Reference			
High school degree	0.92	0.69	0.61	1.38
Some college or technical school	1.71	0.02*	1.11	2.63
College degree or higher	0.83	0.53	0.46	1.48
Household composition				
Alone	Reference			
Partner only	1.04	0.88	0.64	1.70
Partner and children	1.84	0.01*	1.17	2.88
Children only	1.46	0.22	0.79	2.67
Other	0.91	0.83	0.38	2.15
Employment				
Full-time	Reference			
Part-time	0.49	0.00*	0.33	0.73
Seasonal	0.37	0.00*	0.23	0.61
Unemployed/self-employed	0.51	0.14	0.21	1.25
Lives on a Reservation (vs not)	0.74	0.09	0.52	1.05
Length of COVID-19 symptoms				
No COVID-19	Reference			
Less than 2 weeks	1.70	0.23	0.71	4.04
2–3 weeks	3.42	0.00*	1.71	6.85
4 or more weeks	5.18	0.00*	2.06	13.04
Know someone with long COVID (vs not)	1.45	0.02*	1.06	2.00

* p ≤ 0.05

**Table 3:** Multivariate logistic regression of factors associated with not feeling back to normal after COVID-19 infection

	Odds Ratio	p-value	95% Confidence Interval	

Increasing age (by decade)	0.68	0.57	0.18	2.57
Male (vs female)	0.03	0.01*	0.00	0.38
Household composition				
Alone	Ref			
Partner only	0.42	0.56	0.02	7.48
Partner and children	0.30	0.31	0.03	3.06
Children only	30.70	0.02*	1.67	565.35
Other	-			
Lives on Reservation (vs not)	0.98	0.99	0.03	35.76
4 + weeks of COVID symptoms	14.36	0.01*	1.84	111.99

* p ≤ 0.05

**Table 4:** Factor analysis of long COVID symptoms

	Loadings		
Variable	Factor1	Factor2	Factor3

Breathing problems	**0.38**	−0.36	0.11
Altered taste or smell	0.21	−0.28	**0.30**
Thinking problems	−0.10	**0.51**	−0.14
Heart problems	0.04	**0.23**	0.10
Dizziness	0.10	0.20	**0.47**
Abdominal problems	0.07	**0.46**	0.06
Muscle problems	**0.57**	−0.12	−0.06
Altered feelings	**0.47**	−0.13	−0.01
Mood problems	0.18	**0.22**	−0.28
Sleeping problems	**0.47**	−0.18	−0.24
Skin problems	**0.62**	0.39	−0.01
Joint problems	**0.53**	−0.04	−0.19
Headaches	**0.55**	0.24	0.24

**Table 5:** Correlation matrix of long COVID symptoms

	1	2	3	4	5	6	7	8	9	10	11	12	13

Breathing problems	1												
Altered taste or smell	0.27	1											
Thinking problems	−0.21	−0.19	1										
Heart problems	−0.08	0.06	0.09	1									
Dizziness	−0.01*	0.12	0.00*	0.13	1								
Abdominal problems	−0.09	−0.18	0.36	−0.01*	0.23	1							
Muscle problems	0.33	0.05*	−0.05*	0.02*	0.10	−0.01*	1						
Altered feelings	0.30	0.11	−0.12	−0.07	0.04*	0.13	0.33	1					
Mood problems	0.00*	−0.11	0.17	0.19	−0.09	−0.03*	0.05*	0.11	1				
Sleeping problems	0.17	0.14	−0.09	−0.13	−0.10	0.02*	0.38	0.25	0.12	1			
Skin problems	0.04*	0.07	0.11	0.16	0.09	0.17	0.30	0.14	0.25	0.20	1		
Joint problems	0.15	0.05*	−0.10	0.02*	−0.11	−0.02*	0.28	0.23	0.06	0.33	0.39	1	
Headaches	0.15	0.12	0.01*	0.07	0.23	0.08	0.18	0.22	0.07	0.12	0.55	0.28	1

* p ≤ 0.05

**Table 6:** Association between symptom cluster and survey respondent characteristics

	Breathing/muscle/sleeping				Concentration/mood				Smell/dizziness			
	Coef	P > t	[95% Conf	Interval]	Coef	P > t	[95% Conf	Interval]	Coef	P > t	[95% Conf	Interval]

Age category (ref: 18–24)												
25–34	0.13	0.00*	0.05	0.20	−0.05	0.26	−0.14	0.04	−0.04	0.61	−0.19	0.11
35–44	−0.08	0.03*	−0.16	−0.01	−0.13	0.01*	−0.22	−0.03	0.11	0.15	−0.04	0.27
45 or older	−0.07	0.58	−0.31	0.18	−0.05	0.74	−0.35	0.25	0.30	0.23	−0.19	0.80
Male (ref: female)	0.07	0.05*	0.00	0.13	−0.15	0.00*	−0.23	−0.07	0.06	0.35	−0.07	0.20
Household composition (ref: living alone)												
Partner only	0.12	0.01*	0.03	0.22	0.20	0.00*	0.08	0.32	0.23	0.02*	0.04	0.42
Partner and children	0.16	0.00*	0.07	0.24	0.39	0.00*	0.29	0.49	0.27	0.00*	0.11	0.44
Children only	0.06	0.21	−0.04	0.17	0.12	0.05*	0.00	0.25	0.24	0.02*	0.03	0.45
Other	0.08	0.14	−0.03	0.18	0.15	0.02*	0.02	0.28	0.19	0.08	−0.02	0.40
Education (ref: less than high school degree)												
High school degree	0.04	0.53	−0.09	0.16	−0.10	0.19	−0.26	0.05	−0.11	0.41	−0.36	0.15
Some college or technical school	0.08	0.22	−0.05	0.21	−0.06	0.48	−0.22	0.10	−0.18	0.18	−0.44	0.08
College degree or higher	0.04	0.58	−0.10	0.17	−0.24	0.01*	−0.40	−0.07	−0.13	0.34	−0.41	0.14
Lives on a Reservation	0.05	0.38	−0.06	0.17	0.15	0.04*	0.01	0.29	0.04	0.73	−0.19	0.27
Employment (re: full-time)												
Part time	0.24	0.00*	0.15	0.34	0.01	0.85	−0.10	0.12	−0.01	0.92	−0.20	0.18
Seasonal	0.01	0.89	−0.13	0.15	−0.01	0.94	−0.18	0.17	−0.11	0.46	−0.40	0.18
Unemployed/self-employed	0.15	0.01*	0.04	0.26	−0.15	0.04*	−0.28	−0.01	−0.02	0.89	−0.24	0.21
COVID lasting 4 + weeks	0.04	0.21	−0.02	0.11	0.06	0.20	−0.03	0.14	0.03	0.69	−0.11	0.17

* p ≤ 0.05

**Table 7:** Supplemental Great Plains Area American Indian Demographics

Demographics	n (%)

**Age**	
*15–24*	21,259 (27.8)
*25–34*	15,683 (20.5)
*35–44*	13,059 (17.1)
*45–54*	12,503 (16.4)
*55–64*	7,975 (10.4)
*65–74*	3,881 (5.08)
*75–84*	1,620 (2.12)
*85* +	442 (0.58)
**Gender**	
*Female*	59,441 (51.7)
*Male*	55,363 (48.3)
**Education**	
*Less than high school*	11,344 (20.0)
*High School Graduate/Some College*	31,732 (56.1)
*Undergraduate Degree*	11,426 (20.2)
*Graduate or Professional Degree*	2,093 (3.70)
**Unemployment Rate**	12,898 (11.7)
**Uninsured**	35,464 (31.0)

Data from GPTLHB Great Plains Area Demographics^30^. This table includes demographic information for American Indians in the Great Plains Area (South Dakota, North Dakota, Nebraska, and Iowa).

## Data Availability

Data is not available according to established Tribal sovereignty practices.
