# Supplementary material for: The Experience of Long COVID Among American Indian Individuals in Three Great Plains Communities
Source: J Racial Ethn Health Disparities. 2025 Sep 4;13(6):4729–38. doi: 10.1007/s40615-025-02618-z (PMC12933522; doi:10.1007/s40615-025-02618-z)
Supplement: Supplementary file 1 [file 40615_2025_2618_MOESM1_ESM.docx]

The Experience of Long COVID Among American Indian Individuals in Three Great Plains Communities

Journal of Racial and Ethnic Health Disparities

Matthew Tobey, Sara J. Purvis ([spurvis@mgh.harvard.edu](mailto:spurvis@mgh.harvard.edu)), Bethany-Rose Daubman, Mary J. Isaacson, Tinka Duran, Gina Johnson, J.R. LaPlante, Katrina Armstrong.

Survey Questions

Have you heard of long COVID before?

Long COVID is sometimes called Chronic COVID, Long-haul COVID, or post-COVID conditions.

- Yes
- No
- Don't Know

[section break]

Some people who have been infected with the virus that causes COVID-19 can experience long-term effects from their infection, known as post-COVID conditions (PCC) or long COVID. Most people with COVID-19 get better within a few days to a few weeks after getting sick, so at least 4 weeks after getting sick is just the start of when post-COVID conditions could first be identified.

Do you know anyone within your community, family, or friends that have had these symptoms or that have had long COVID?

- Yes
- No

Is long COVID a major issue in your community?

- Yes
- No

For the next questions, if you have had COVID-19 more than once, please answer based on your worst case of

COVID-19.

How would you describe your COVID-19 symptoms when they were at their worst?

- I had no symptoms
- I had mild symptoms
- I had moderate symptoms
- I had severe symptoms

Are you back to normal after having COVID-19 or are you still having symptoms?

- Yes, I am back to normal
- No, I still have some or all of my symptoms

How long did you have COVID-19 symptoms overall? Please include time spent with mild symptoms and the

time in between symptoms if these have been coming and going.

Symptoms may include: tiredness or fatigue; difficulty thinking, concentrating, forgetfulness, or memory

problems (sometimes referred to as "brain fog"); difficulty breathing or shortness of breath; joint or

muscle pain; fast-beating or pounding heart (also known as heart palpitations); chest pain; dizziness

when standing; menstrual changes; changes to taste/smell; or inability to exercise.

- Less than 2 weeks
- 2-3 weeks
- 4-7 weeks
- 8-12 weeks
- More than 12 weeks

Did these long-term symptoms reduce your ability to carry out day-to-day activities compared with the time before you had COVID-19?

- Not at all
- Yes, a little
- Yes, a lot

For how long were you unable to function as normal due I was always able to function as normal to COVID-19 symptoms?

- 1-3 days
- 4-6 days
- 7-13 days
- 2-3 weeks
- 4-7 weeks
- 8-12 weeks
- 12+ weeks

How many days were you or have you been so unwell that you stayed in bed or on the couch after getting sick with COVID-19?

None

- 1-3 days
- 4-6 days
- 7-12 days
- 2-3 weeks
- 4-7 weeks
- 8-12 weeks
- 12+ weeks

Did you have any of the following problems 12 weeks (3 months) or more after getting sick with COVID-19? Please only select symptoms that were not explained by another reason.

- I was back to my usual self.
- Breathing problems (e.g., breathlessness, pain on breathing, cough)
- Altered sense of taste or smell
- Problems thinking and communicating (e.g., brain fog, memory problems, difficulty concentrating, decreased alertness, confusion, difficulty speaking)
- Heart problems (e.g., chest pain, palpitations)
- Light-headedness/dizziness on standing
- Abdominal problems (e.g., tummy pain, diarrhea, appetite loss)
- Muscle problems (e.g., muscle aches, weakness, severe fatigue)
- Altered feelings in your body (unusual tingling, pain)
- Problems relating to mood (e.g., anxiety, feeling 'down', or irritable)
- Problems sleeping (e.g., poor sleep or excessive sleep)
- Skin rashes
- Bone or joint pain
- Headaches

Did you have any extra difficulty with any of the following activities 12 weeks (3 months) after you became sick with COVID-19? Please select all that apply.

- Learning a new task (e.g., learning how to get to apply. a new place)
- Standing for long periods, such as 30 minutes
- Taking care of your household responsibilities
- Joining on community activities (e.g., festivities, religious, other)
- Being emotionally affected by your health problems
- Concentrating on doing something for 10 minutes
- Walking a long distance, such as a half a mile
- Washing your whole body
- Getting dressed
- Dealing with people that you do not know
- Maintaining a friendship
- Your day-to-day work/school (includes paid & unpaid work)
